# Dsi-RNA knockdown of genes regulated by Foxo reduces glycogen and lipid accumulations in diapausing *Culex pipiens*

**DOI:** 10.1038/s41598-020-74292-6

**Published:** 2020-10-14

**Authors:** Olatunde P. Olademehin, Chengyin Liu, Binayak Rimal, Nathaniel F. Adegboyega, Fu Chen, Cheolho Sim, Sung Joon Kim

**Affiliations:** 1 Department of Chemistry and Biochemistry, Baylor University, One Bear Place #97348, Waco, TX 76706 USA; 2 Department of Chemistry, Howard University, 525 College St. N.W., Washington, D.C. 20059 USA; 3 Institute of Biomedical Studies, Baylor University, One Bear Place #97348, Waco, TX USA; 4 Department of Environmental Sciences, Southern Illinois University Edwardsville, Edwardsville, IL 62026 USA; 5 Department of Chemistry and Biochemistry, University of Maryland, College Park, MD 20742 USA; 6 Department of Biology, Baylor University, One Bear Place #97348, Waco, TX 76706 USA

**Keywords:** Polysaccharides, Solid-state NMR

## Abstract

*Culex pipiens* is a major carrier of the West Nile Virus, the leading cause of mosquito-borne disease in the continental United States. *Cx. pipiens* survive overwinter through diapause which is an important survival strategy that is under the control of insulin signaling and Foxo by regulating energy metabolism. Three homologous candidate genes, *glycogen synthase* (*glys*), *atp-binding cassette transporter* (*atp*)*,* and *low-density lipoprotein receptor chaperone* (*ldlr*), that are under the regulation of Foxo transcription factor were identified in *Cx. pipiens*. To validate the gene functions, each candidate gene was silenced by injecting the target dsi-RNA to female *Cx. pipiens* during the early phase of diapause. The dsi-RNA injected diapause-destined female post-adult eclosion were fed for 7 days with 10% glucose containing 1% d-[^13^C_6_]glucose. The effects of dsi-RNA knockdown on glucose metabolism in intact mosquitoes were monitored using ^13^C solid-state NMR and ATR-FTIR. Our finding shows that the dsi-RNA knockdown of all three candidate genes suppressed glycogen and lipid biosyntheses resulting in inhibition of long-term carbon energy storage in diapausing females.

## Introduction

*Culex pipiens* is the mosquito that vectors the West Nile Virus, St Louis and equine encephalitis viruses, bird malaria, and dog heartworms in North America. In late summer and early winter, this mosquito, like some other insects, initiates a diapause program in response to short photoperiods and decreasing temperatures^1^ The diapausing females do not seek blood but instead seek flower or fruit nectars, primarily consisting of glucose, fructose, and sucrose^2^, to convert mono and disaccharides into glycogen and fat. This metabolic shift doubles its fat and glycogen reserves, which are used as an energy source for survival during the long winter season. During the first month of diapause glycogen reserve is consumed only as an energy source, and after glycogen is used up the energy demands are fueled from fat in the fat body. Thus, fat and glycogen storage in early diapause and regulation of their timely consumption are critical features for successful overwintering.

Insulin/Foxo signaling is a key component of the cascade regulating early diapause energetics in *Cx. pipiens*^3^. The insulin signal is not activated in the early diapause program and subsequently lifting suppression of a forkhead of transcriptional factor (Foxo). The inactivation of the Foxo results in the reduction of nutrient storage, which suggests that the downstream genes targeted by the Foxo are crucial for glycogen and fat homeostasis in diapausing females^3^.

To understand the key genetic components that are involved in the energy storage, ChIP-seq analysis was used to identify three target genes under the control of Foxo, which are relevant to fat and glycogen transport and utilization^4^. Three candidate genes are *glys*, *atp*, and *ldlr*. The *glys* gene encodes for glycogen synthase which is a key enzyme in glycogenesis that catalyzes the conversion of monosaccharide glucose into a polymeric chain of glycogen for storage. In *Cx. pipiens*, glycogen is rapidly accumulated during the early diapause (7–10 days after adult eclosion) and is used to maintain energy homeostasis during the first month of diapause^5^. Although the mechanism that governs differential carbohydrate utilization is unknown, we speculate that the regulation of *glys* is closely involved in glucose metabolism during the early nectar feed. In addition, two genes *atp* and *ldlr* have potential roles in intracellular lipid transport processes and cellular lipid homeostasis^6,7^. These two genes may be linked to the transport of various lipids across membranes to store and distribute nutrients in the diapausing fat body cells. To validate the gene functions, synthetic dicer-substrate siRNAs (dsi-RNA) were used to silence the candidate genes by injecting the target dsi-RNAs to female *Cx. pipiens* during the early phase of diapause.

**Figure 1:**
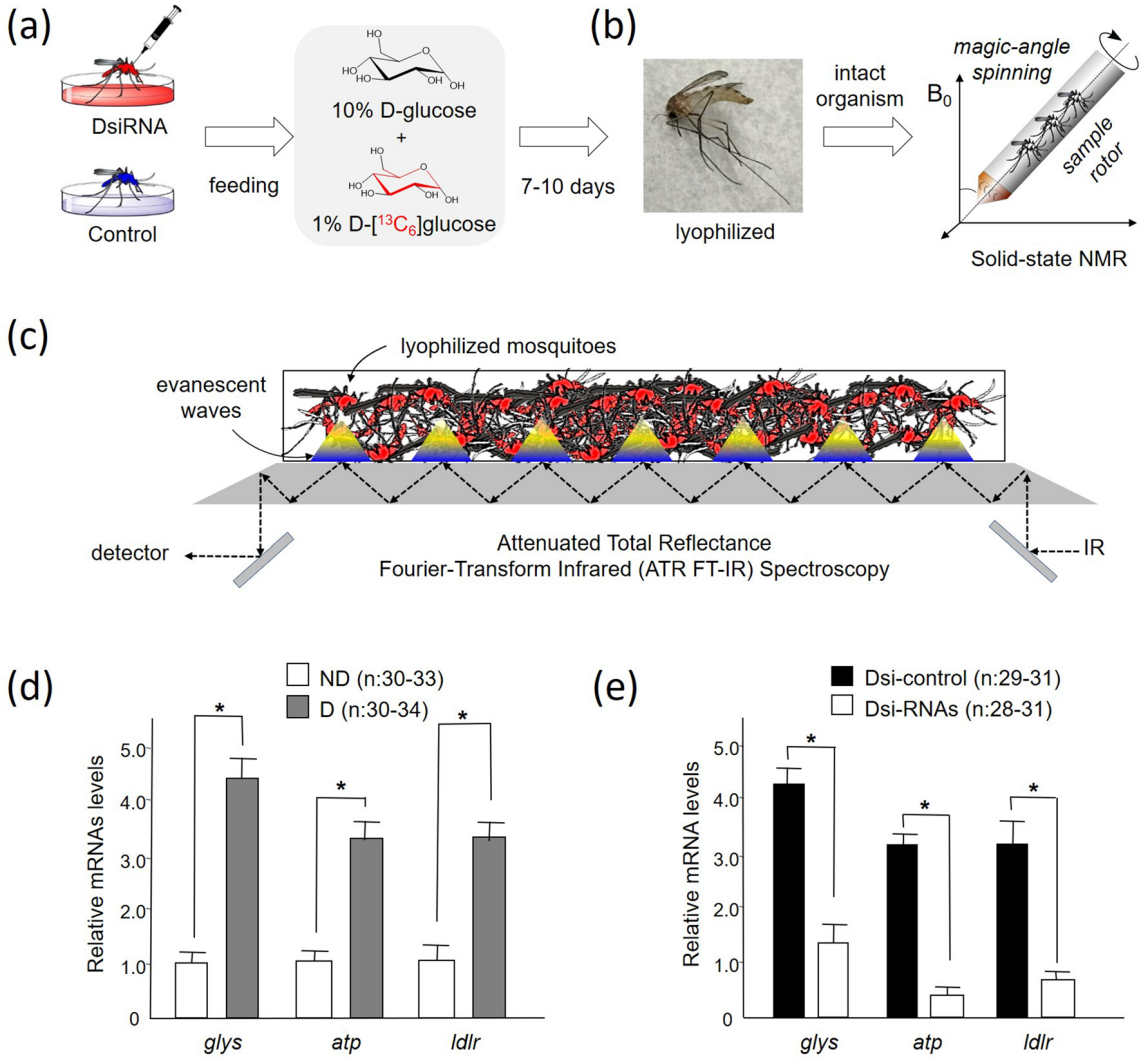
^13^C-Isotope labeling of *Culex pipiens* for solid-state NMR and ATR-FTIR analysis. (**a**) Nondiapausing female *Cx. pipiens* within 1 day after eclosion were injected (0.5 µg/♀) with dsi-*glys* or with dsi-control into the thorax of cold-anesthetized mosquitoes. Then the mosquitoes were fed with 10% glucose containing 1% ^13^C-isotope labeled D-[^13^C_6_]glucose (uniformly ^13^C labeled with 99% isotopic enrichment). After 7 days of feeding, the mosquitoes were frozen then lyophilized. (**b**) The lyophilized ^13^C-labeled intact mosquitoes were placed into a 3.2-mm zirconia rotor and spun at 12 kHz magic-angle spinning for ^13^C solid-state NMR analysis. (**c**) Schematic representation of attenuated total reflectance Fourier transform infrared (ATR-FTIR) spectroscopy for direct quantification of glycogen and lipid accumulations in diapausing female *Cx. pipiens*. (**d**) RNA interference efficiency of synthetic dicer-substrate siRNA (dsi-RNA) treated females of *Cx. pipiens* Quantitative real-time PCR showing expression of glycogen synthase (*glys*), ATP-binding cassette transporter (*atp*), and low-density lipoprotein receptor chaperone (*ldlr*) in *Cx, pipiens* females, 7–10 days after adult eclosion. ND (white), programmed by long day-length for nondiapause; D (gray), programmed by short day-length for diapause. A ribosomal protein large subunit 19 (*rpl19*) is used as a loading control. Bars (mean ± s.e., n=3) with asterisks (*) indicate significant differences at *P* < 0.05, *t* test. (**e**) Transcript levels of *glys, atp,* and *ldlr* in diapausing females injected with dsi-*glys*, dsi-*atp,* or dsi-*ldlr* (white bars) were compared with the dsi-controls (black bars). Expression levels were measured by quantitative real-time PCR 7 days after dsi-RNA injections, which was normalized using *rpl19* loading controls. Bars (mean ± s.d.) with asterisks (*) indicate significant differences at *P* < 0.05, *t* test.

In this study, the effects of three candidate genes knockdown on glucose metabolism were characterized using a combined ^13^C solid-state nuclear magnetic resonance (NMR) and attenuated total reflection Fourier-transform infrared spectroscopy (ATR-FTIR) methods. Solid-state NMR is a powerful technique that enables a direct measure of chemical compositions of intact whole cells^8–10^ and entire organisms^11,12^. Solid-state NMR measurements were carried out on the lyophilized dsi-RNA injected diapause-destined females of *Cx. pipiens* that were fed for 7 days with uniformly ^13^C-labeled d-[^13^C_6_]glucose (Fig. 1a). Since only the ^13^C isotope is NMR active, solid-state NMR was used to measure the newly synthesized ^13^C-labeled glycogen and lipids that were metabolized from the provisioned d-[^13^C_6_]glucose. Then, ATR-FTIR was used to measure the total glycogen and lipid accumulations in mosquitoes (Fig. 1b). ATR-FTIR is a highly sensitive technique that can rapidly quantify protein, lipid, and glycogen compositions of individual mosquitoes (Fig. 1c). Recently, ATR-FTIR has been developed as a portable instrument for the identification and screening of individual *Aedes aegypti* mosquitoes with Wolbachia infection^13^. Unlike ^13^C solid-state NMR which measures the d-[^13^C_6_]glucose utilization, FTIR is independent of the ^13^C-labeling. Thus, FTIR was used to determine the total glycogen and lipid compositions in mosquitoes. By combining FTIR and solid-state NMR measurements, the effects of the candidate gene knockdown on the glucose utilization for the newly synthesized ^13^C-labeled glycogen and lipids, and the changes in the total accumulations of glycogen and lipids in dsi-RNA injected mosquitoes were determined.

## Results and discussion

### Dsi-RNA injection into adult female *Cx. pipiens* and dsi-RNA efficiency by qRT-PCR

mRNA expression levels of the genes *glys*, *atp*, and *ldlr* in nondiapausing (ND) and diapausing (D) *Cx. pipiens* were obtained using quantitative real-time PCR (qRT-PCR). The mRNA expression patterns of these genes showed that all three genes were more than twofold upregulated in diapausing mosquitoes 7–10 days after eclosion compared to nondiapausing mosquitoes reared at short day-length (Fig. 1d). Increased expression of the *glys, atp,* and *ldlr* genes in mosquitoes in response to short day-length (destined to diapause) strongly suggested that these genes may be involved in the nutrient storage for overwintering.

The dsi-RNA efficiency was assessed by qRT-PCR. Compared to the relatively high induction of *glys, atp* and *ldlr* in dsi-control injected mosquitoes, less than 30% of *glys, atp,* and *ldlr* mRNA were detected in dsi-RNA-injected diapausing mosquitoes (Fig. 1e, P < 0.05, *t* test). This confirmed that the injection of dsi-*glys*, dsi-*atp,* and dsi-*ldlr* successfully inhibited the induction of the *glys, atp,* and *ldlr* genes, respectively. The absence of changes in the basal expression of ribosomal protein large subunit 19 in mosquitoes after 7 days post-injection indicated that the observed low levels of *glys, atp,* and *ldlr* genes were related to the knock-down effect of dsi-RNA treatments rather than variation in sample loading.

### Direct quantification of ^13^C-labeled glycogen by solid-state NMR

**Figure 2:**
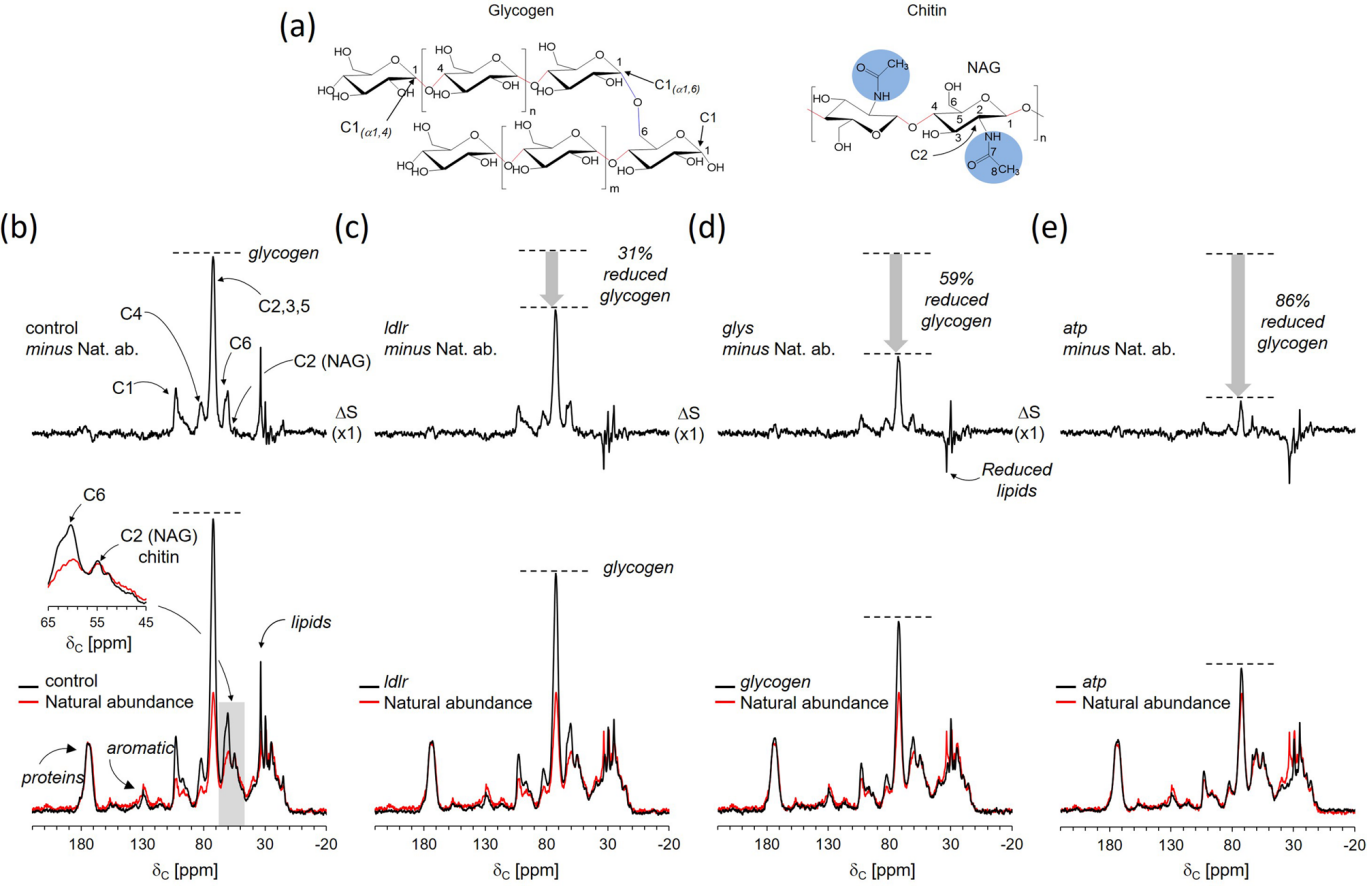
^13^C-CPMAS NMR of diapausing female *Cx. pipiens* fed with d-[^13^C_6_]glucose. (**a**) Chemical structure of glycogen (left) and chitin (right). Glycogen consists of linear d-glucose with *α*(1,4) connection with the branching of *α*(1,6). Chitin is a glycopolymer of repeating *N*-acetyl-glucosamine connected by *α*(1–4) glycosidic linkage. The *N*-acetyl group on chitin is highlighted in blue. (**b**) 125-MHz ^13^C-CPMAS spectrum of diapause bound dsi-control treated mosquitoes that were fed for 7-days post adult eclosion with 1% d-[^13^C_6_]glucose mixed with 10% natural abundance Glc (bottom black). The natural abundance spectrum of untreated mosquitoes (nondiapause) fed with Glc is shown at the figure bottom (red). The difference spectrum (top black) is a result of spectral subtraction of the natural abundance (Nat. ab.) of nondiapause from diapause-destined 1% d-[^13^C_6_]glucose fed mosquitoes. The difference spectrum shows that the d-[^13^C_6_]glucose is primarily routed to glycogen biosynthesis. The figure inset shows the enlarged spectra centered at 55 ppm, which corresponds to the C2 of NAG in chitin, does not increase in mosquitoes that were fed with d-[^13^C_6_]glucose. Therefore, the provisioned d-[^13^C_6_]glucose is not routed to chitin biosynthesis during the diapause. (**c**) ^13^C-CPMAS spectrum of dsi-*ldlr* treated mosquitoes. (**d**) ^13^C-CPMAS spectrum of dsi-*glys* treated mosquitoes. (**e**) ^13^C-CPMAS spectrum of diapause bound dsi-*atp* treated mosquitoes. Storage of d-[^13^C_6_]glucose by glycogen biosynthesis is reduced by 31, 46, and 56% for dsi-*ldlr*, dsi-*glys*, and dsi-*atp*-treated mosquitoes, respectively. Each spectrum is the result of the accumulation of 1024 scans. The magic angle spinning was at 10 kHz.

The effects of *glys, atp,* and *ldlr* knockdown on the uptake and utilization of d-[^13^C_6_]glucose for glycogen and lipid biosyntheses were determined using solid-state NMR (Fig. 1a). 125-MHz ^13^C-CPMAS spectra of dsi-RNA-injected mosquitoes are shown in Fig. 2(b–e, bottom black). Each spectrum is shown with overlapping ^13^C-natural abundance spectrum (red) of diapause-destined females without the dsi-RNA injection that was fed for 7 days with 10% sucrose. The carbon chemical shift assignments for the observed resonances are as follow: 175 ppm for peptidyl-carbonyl carbons in proteins, 130 ppm for aromatic and ethylene carbons in nucleic acids and lipid head groups, 60–105 ppm for the *O*-alkyl carbons in carbohydrates, and 10–40 ppm for the aliphatic carbons of lipids. The spectra were normalized to the 175-ppm intensity for comparison.

**Figure 3:**
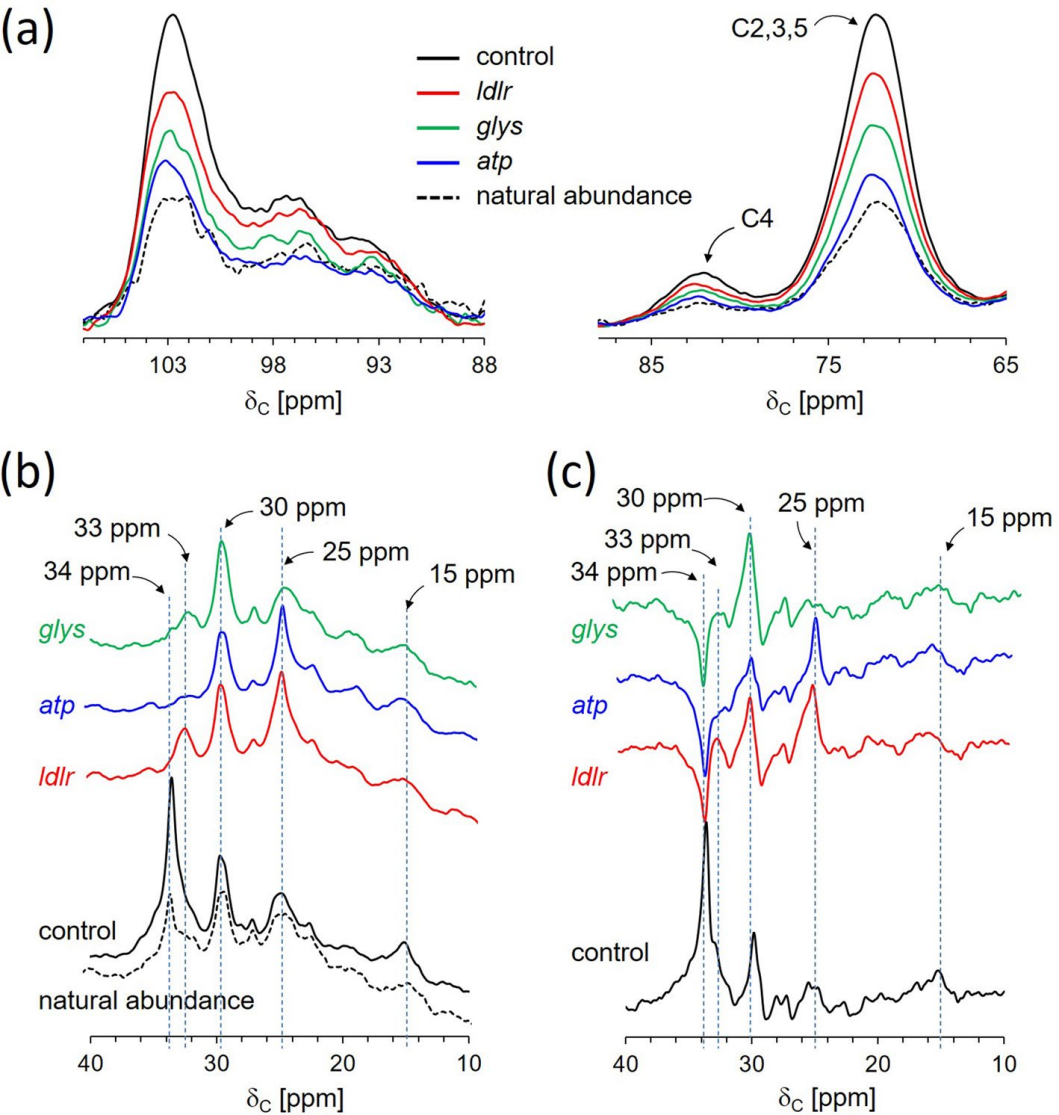
Reduced glycogen and lipid accumulations in diapausing female *Cx. pipiens* by solid-state NMR. (**a**) Overlapping ^13^C-CPMAS spectra centered at 96 ppm of C1 anomeric carbon (left) and 75 ppm for the C2, C3, C4, and C5 carbons in glucose (right). The dsi-RNA knockdown of all three candidate genes under the control of Foxo show suppressed glycogen biosynthesis in diapausing mosquitoes. (**b**) Enlarged ^13^C-CPMAS spectra of dsi-RNA knockdown mosquitoes show the disappearance of aliphatic –CH_2_– carbons at 34 ppm and accumulation at 30 and 25 ppm. (**c**) The reduced 34-ppm peak intensity, which is clearly visible in the difference spectra (natural abundance removed), indicates reduced lipid accumulation in dsi-RNA knockdown mosquitoes.

All ^13^C-CPMAS spectra of d-[^13^C_6_]glucose-fed diapausing mosquitoes show intense *O*-alkyl carbon resonances at 61, 73, 82, 93, 99, and 104 ppm (Fig. 2). The 61-ppm peak is assigned to the C6 of glucose, 73 ppm to the C2, C3, and C5 carbons, and 84 ppm to C4 of glucose. The anomeric carbon at the C1 position has chemical shifts in the range of 90–110 ppm depending on the glycosidic linkages. The CPMAS spectra are consistent with the routing of d-[^13^C_6_]glucose in diapausing mosquitoes for glycogen biosynthesis, but not to chitin biosynthesis. In the event of chitin biosynthesis, the d-[^13^C_6_]glucose needs to be converted to *N*-acetylglucosamine (GlcNAc). This results in the change of C2 chemical shift at 73 ppm in glucose to 55 ppm in GlcNAc^14^. The absence of an increase in 55-ppm peak intensity (Fig. 2b) strongly supports that the uptake of glucose during diapause is exclusively routed to glycogen biosynthesis^15^. Thus, the changes in the 73-ppm and 103-ppm peak intensities are directly proportional to the amount of ^13^C-labeled glycogen accumulations in mosquitoes (Fig. 3a). The largest glycogen accumulation was observed for the control mosquitoes injected with *β-gal* dsi-RNA, referred to as dsi-control, followed by the dsi-*ldlr*, dsi-*glys*, and dsi-*atp* injected mosquitoes.

To accurately quantify the ^13^C-labeled glycogen accumulation in mosquitoes, the ^13^C-natural abundance contribution was removed by spectral subtraction of the natural abundance ^13^C-CPMAS spectrum of dsi-RNA treated mosquitoes. In the difference spectrum (Fig. 2b–e, top) intense *O*-alkyl carbon resonances of glycogen are visible. The maximum glycogen accumulation was observed for the dsi-control, followed by the dsi-*ldlr* with 31% reduction, dsi-*glys* with 59% reduction, and dsi-*atp* injected mosquitoes with 86% reduction with respect to the dsi-control (Fig. 2c–e). Since the dsi-RNA knockdown of the genes *glys*, *atp*, and *ldlr* have all negatively impacted the d-[^13^C_6_]glucose utilization for the biosynthesis of glycogen, this strongly indicates that these genes are directly involved in the long-term carbon storage for the energy homeostasis in diapause programmed female mosquitoes.

### Direct quantification of ^13^C-labeled lipids by solid-state NMR

The ^13^C-CPMAS spectrum of the dsi-control (Fig. 3b) shows increases in the CH_2_ carbon intensities at 34, 33, 30, and 25 ppm, and CH_3_ at 15 ppm with the most predominant increase observed at 34 ppm, which is consistent with the utilization of d-[^13^C_6_]glucose for lipid biosynthesis. However, the 34-ppm peak in the CPMAS spectra of dsi-RNA injected mosquitoes targeting Foxo downstream elements is absent (Fig. 3b) and appears as a negative intensity in the difference spectra (Fig. 3c). The negative aliphatic carbon intensity indicates that the dsi-RNA knockdown of *ldlr*, *atp*, and *glys* inhibited the routing of d-[^13^C_6_]glucose for de novo lipid biosynthesis while an increase in the metabolism of the stored lipids in mosquitoes. Interestingly, the reduction of 34-ppm intensity was compensated by increases in 30 and 25-ppm intensities (Fig. 3c), which suggests a complex change in lipid composition that is associated with the knockdowns.

### FTIR absorption band assignments

**Figure 4:**
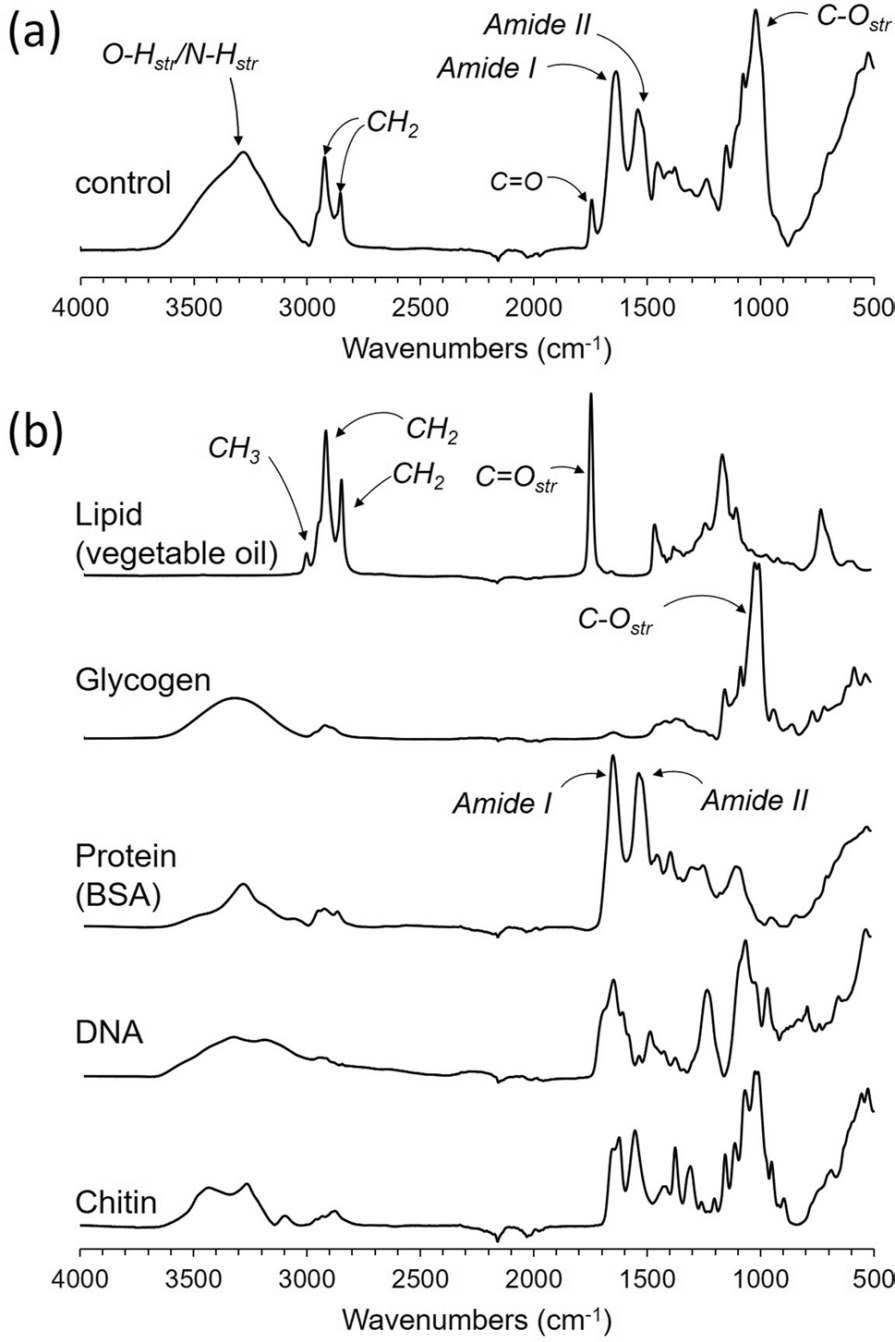
ATR-FTIR spectra of diapausing female *Cx. pipiens* and chemical components. (**a**) FTIR spectrum of dsi-control treated diapausing female *Cx. pipiens* were fed with 10% Glc for 7-days post adult eclosion. (**b**) FTIR spectra of representative chemical composition in mosquitoes: lipids, glycogen, proteins, DNA, and chitin.

**Figure 5:**
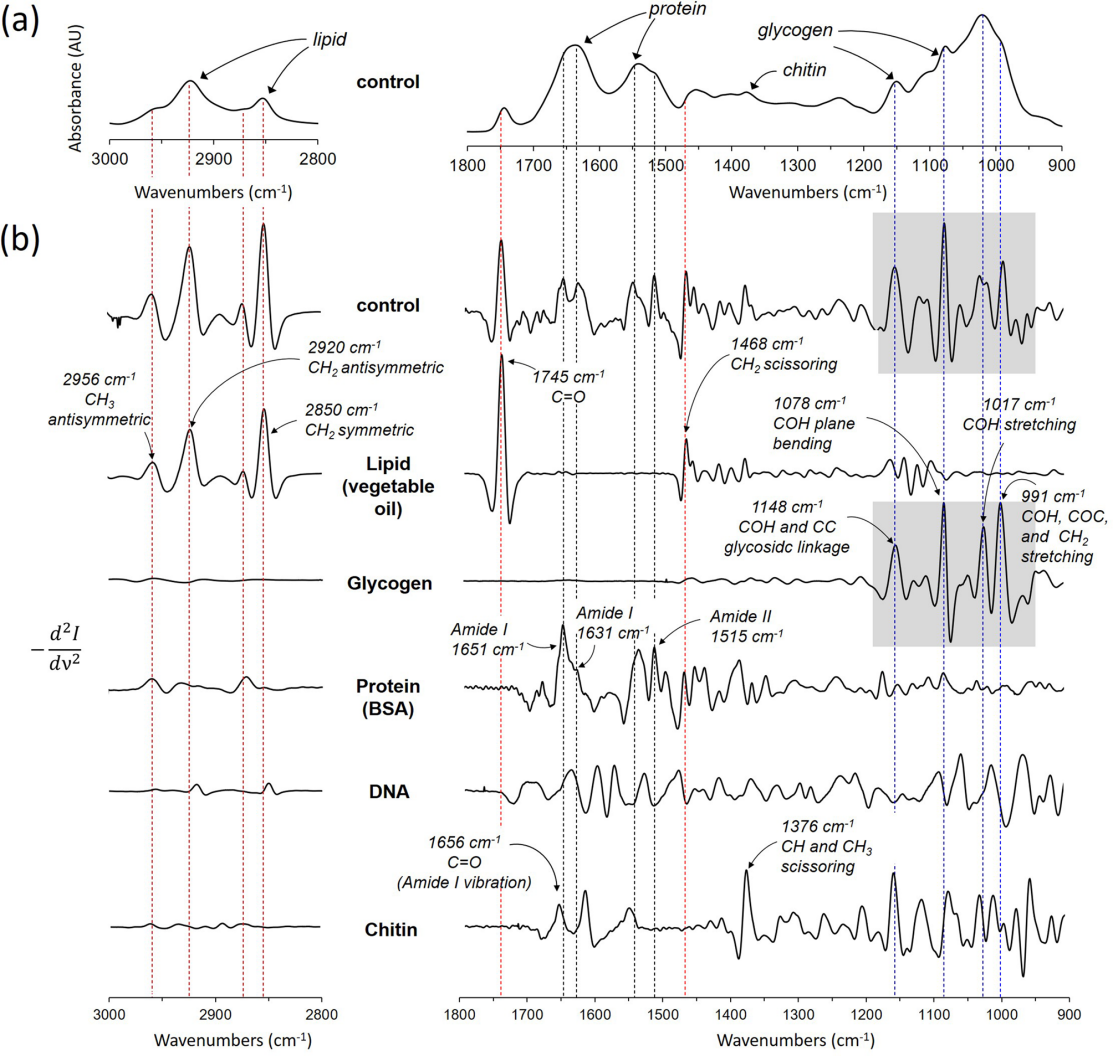
FTIR absorption band assignment. (**a**) FTIR spectrum of dsi-control treated diapause female *Cx. pipiens* were fed with 10% Glc for 7-days post adult eclosion. (**b**) Second-order derivative FTIR spectra of dsi-control treated diapausing females of *Cx. pipiens*, lipid (vegetable oil), glycogen, protein (BSA), DNA, and chitin. The red dotted lines mark the absorption bands 2956, 2920, 1745, and 1468 cm^−1^ of lipids, the black dotted lines mark 1651, 1631, 1515 cm^−1^ of amide I and II found in proteins, and the blue dotted lines for 1148, 1078, 1017, and 991 cm^−1^ of glycogen.

Total lipid, protein, and glycogen accumulation in lyophilized mosquitoes were monitored using ATR-FTIR. IR absorption spectrum of lyophilized dsi-control injected diapausing *Cx. pipiens* is shown in Fig. 4a, along with the IR spectra for the representative cellular components (Fig. 4b): lipid, glycogen, protein, DNA, and chitin. The characteristic IR absorption bands for each lipid, glycogen, protein (BSA), DNA, and chitin were identified by taking a second-order derivative of the absorption spectra (Fig. 5). A second-order derivative (Fig. 5) removes the broad component, revealing the narrow absorption bands from the spectrum. The characteristic absorption bands for lipids are 2956 cm^−1^, 2850 cm^−1^, and 1745 cm^−1^. The IR assignments for 2956 cm^−1^ is for CH_3_ antisymmetric stretch, 2920 cm^−1^ for CH_2_ antisymmetric stretch, 2850 cm^−1^ for CH_2_ symmetric, 1745 cm^−1^ for C=O stretch, and 1468 cm^−1^ for CH_2_ scissoring^16^. These absorption bands are predominant in vegetable oil but absent from other representative cellular components. For protein (bovine serum albumin), Amide I at 1651 and 1631 cm^−1^, which appear as the most intense absorption bands are assigned to C=O, C–C, and C–N stretching of the protein-peptide backbone. The Amide II, positioned at 1510–1580 cm^−1^, are assigned to in-plane N–H bending vibrations, and C-N and C–C stretching vibrations. Amide I and II bands (1651, 1631, and 1515 cm^−1^) are visible in the second-order derivative spectrum of the dsi-control treated mosquitoes but are absent from the derivative spectra of lipid, glycogen, DNA, and chitin. The IR absorption band assignments for the second-order derivative spectrum of glycogen^17^ are as follow: (i) 991 cm^−1^ is assigned to in-plane bendings of CH_2_, and COH, and CO and COC stretching of glycosidic linkage, (ii) 1017 cm^−1^ for COH stretching, (iii) 1078 cm^−1^ for in-plane bending of COH, and (iv) 1149 cm^−1^ for COC and CC stretching modes of glycosidic linkage and asymmetric ring stretching. Despite the broad overlapping IR absorption bands of glycogen and chitin (Fig. 4b), the second-order derivative spectra of dsi-control treated mosquitoes at 950–1800 cm^−1^ range (Fig. 7d) closely resembles glycogen (Fig. 5, gray boxes) and not chitin.

### Total lipid quantification by FTIR

**Figure 6:**
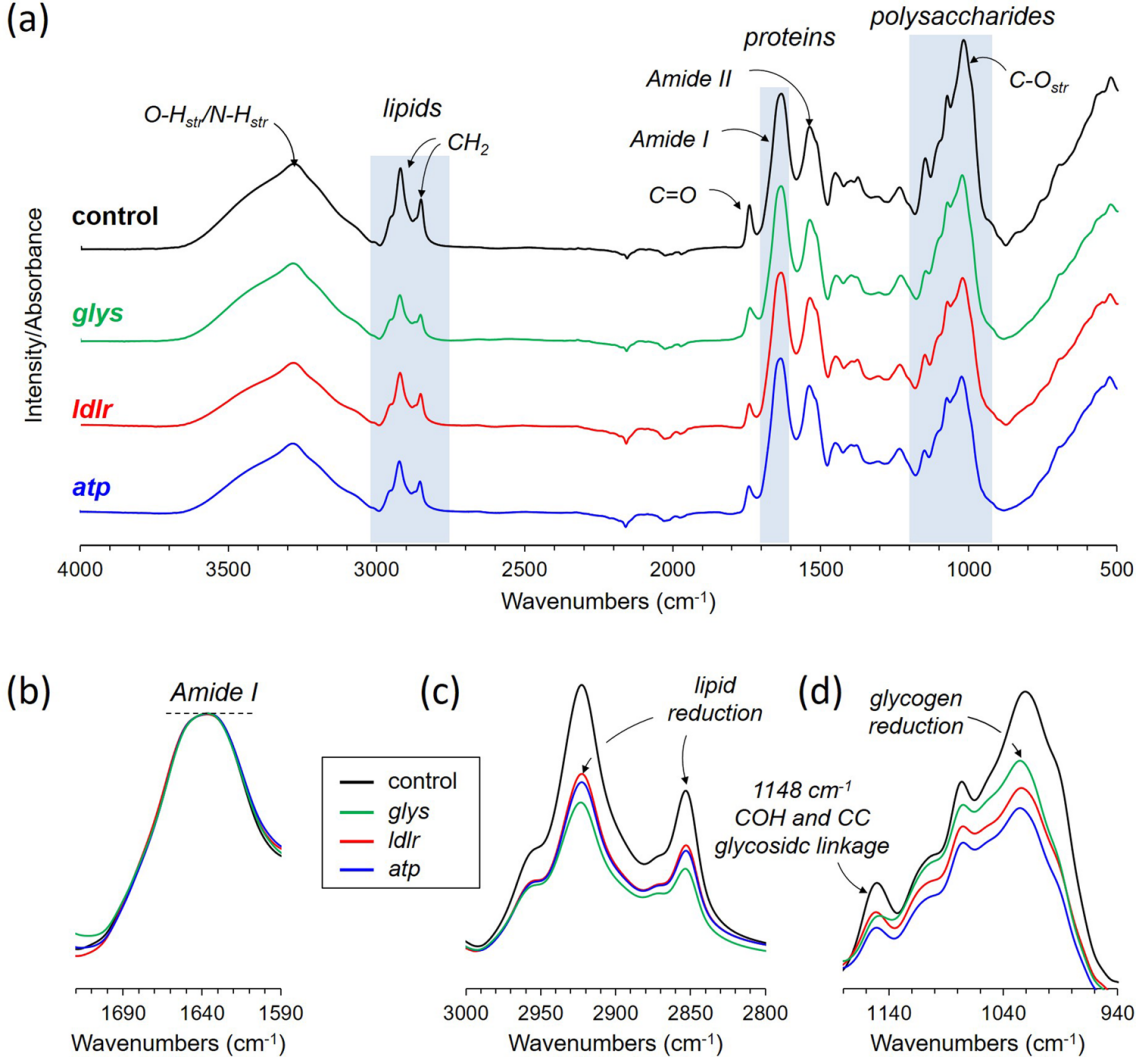
Direct quantification of total glycogen and lipid in *Cx. pipiens* by FTIR. (**a**) ATR-FTIR spectra of dsi-control, dsi-*glys*, dsi-*ldlr*, and dsi-*atp*-treated diapausing females of *Cx. pipiens* fed with 10% Glc for 7-days post adult eclosion. (**b**) The FTIR spectra were normalized to the amide I intensity at 1631 cm^−1^. (**c**) The absorption of lipid 2800–3000 cm^−1^ shows a large reduction of lipid accumulations in mosquitoes that were treated with dsi-RNAs that targeted the downstream elements of Foxo. (**d**) Polysaccharide regions show reduced glycogen accumulations in dsi-RNA treated mosquitoes.

**Figure 7:**
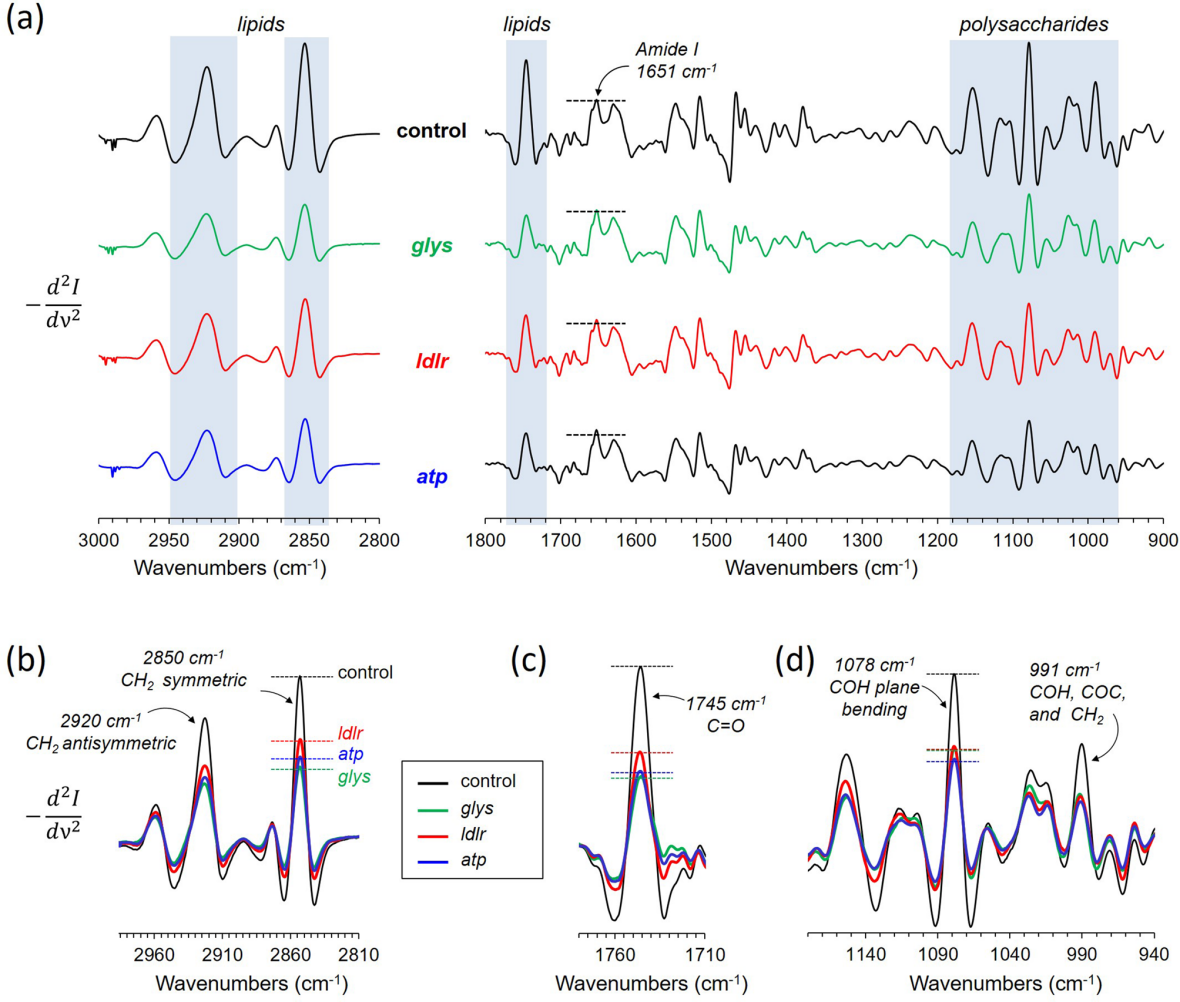
Second-order derivative FTIR spectra of diapausing female *Cx. pipiens*. (**a**) Second-order derivative FTIR spectra of dsi-RNA treated female *Cx. pipiens* from Fig. 6a. (**b**) Overlapping second-order derivative FTIR spectra of mosquitoes enlarged for lipid absorption bands at 2920 and 2850 cm^−1^. (**c**) Derivative spectra centered at 1745 cm^−1^ for C=O stretch found in lipids (Fig. 5b). (**d**) Second-order derivative spectra centered at 1078 cm^−1^ for in-plane bending of COH found in glycogen.

FTIR spectra of lyophilized diapausing females treated with dsi-RNAs (dsi-control, dsi-*glys*, dsi-*atp*, and dsi-*ldlr*) and its second-order derivative spectra are shown in Figs. 6a and 7a, respectively, with the characteristic IR absorption bands representing the total protein, lipid, and glycogen highlighted in blue boxes. Both FTIR spectra and the second-order derivative are normalized to Amide I intensity at 1631 cm^−1^ (Figs. 6b and 7a), which is almost entirely due to C=O stretching vibrations. The normalization assumes that the molar absorptivity of C=O stretching vibration for each protein secondary structural element is essentially the same^18^. The Amide I normalization of FTIR spectra is comparable to the normalization of 175-ppm peak intensity in the ^13^C-CPMAS spectra of Fig. 2. Since the uptake of glucose by mosquitoes during the diapause is not routed to protein or chitin biosynthesis, the total amount of protein in individual mosquito during the early window of diapause remains constant.

Enlarged IR spectra of dsi-RNA injected diapausing females for the characteristic IR absorption bands for lipids at 2920 cm^−1^ and 2850 cm^−1^ are shown in Fig. 6c and its corresponding second-order derivative spectra in Fig. 7b. The total lipids in mosquitoes were estimated based on the IR absorption intensity of CH_2_ at 2920 cm^−1^. The maximum lipid accumulation was observed for dsi-control mosquitoes followed by dsi-*ldlr* (33% reduction), dsi-*atp* (36% reduction), and dsi-*gly*s injected mosquitoes (44% reduction). The total triacylglyceride (TAG) accumulation in mosquitoes was also determined by comparing the intensity of absorption at 1745 cm^−1^. The IR band at 1745 cm^−1^ which is also found in the IR spectrum of vegetable oil (Fig. 4b) is assigned to the C=O stretch of the ester-linked carbons found in glycerol of TAG. Since intense Amide I at 1651 cm^−1^ overlaps significantly with the 1745 cm^−1^, the second-order derivative of the spectra was used to remove the broad component. The resolved narrow C=O stretch band at 1745 cm^−1^ (Fig. 7c) was used for comparison. The maximum TAG accumulation was observed for dsi-control followed by dsi-*ldlr*, dsi-*atp*, and dsi-*gly*s injected mosquitoes. Hence, the dsi-RNA knockdown of the genes *glys*, *ldlr*, and *atp* resulted in the reduction of TAG (lipids) in diapausing mosquitoes.

It is important to point out that the FTIR measurements were performed on powdered lyophilized mosquitoes, a group that consisted of 40–60 individuals. The use of a powdered lyophilized sample was critical because of the high sensitivity of the FTIR instrument which can readily differentiate the chemical composition based on the different body segments. Thus, the FTIR spectra of an intact individual mosquito can vary significantly based on the position of the body segment where the measurements were taken. The use of the powdered mosquitoes eliminated the spectral variations associated with the sampling.

### Total glycogen quantification by FTIR

For glycogen analysis, the overlapping IR absorption bands between 940 and 1180 cm^−1^ (Fig. 6d) are assigned to the stretching and bending vibrational modes of COH, and CO and COC found in polysaccharides. Although the IR absorption bands found in glycogen and chitin overlap significantly (Fig. 4), the second-order derivative spectra (Fig. 7d) clearly resolves 1078 cm^−1^ (COH plane bending) and 991 cm^−1^ (COH, COC, and CH_2_ stretching) bands unique to glycogen. Also, since glucose uptake during the diapause is not routed to chitin biosynthesis, as determined by solid-state NMR (Fig. 2b), the IR absorption intensity at 1017 cm^−1^ (Fig. 6d) is directly proportional to the total glycogen accumulation in mosquitoes. The maximum glycogen accumulation was observed for the dsi-control, followed by dsi-*gly*s, dsi-*ldlr*, and dsi-*atp*. Thus, the dsi-RNA knockdown of the genes *glys*, *ldlr*, and *atp* showed a significant reduction in total glycogen accumulation in diapausing mosquitoes.

The order of total glycogen accumulations from the most to least as measured by FTIR was dsi-control, followed by dsi-*glys*, dsi-*ldlr*, and dsi-*atp* injected mosquitoes (Fig. 6d). This differed from solid-state NMR which measured the maximum ^13^C-labeled glycogen accumulation was dsi-control, followed by dsi-*ldlr*, dsi-*glys*, and dsi-*atp* injected mosquitoes (Figs. 2 and 4a). The solid-state NMR data show that the newly synthesized ^13^C-labeled glycogen accumulation in dsi-*glys* was approximately 41% less than the dsi-*ldlr* injected mosquitoes, but the total glycogen in dsi-*glys* as measured by the FTIR was greater than dsi-*ldlr* injected mosquitoes. This indicates that the dsi-*glys* knockdown primarily suppressed the d-[^13^C_6_]glucose utilization for the de novo glycogen biosynthesis without a significant impact on the total glycogen (Fig. 6d, green line). In contrast, the dsi-*ldlr* injected mosquitoes show increased accumulation of ^13^C-labeled glycogen but a decrease in total glycogen (Fig. 6d, red line). Hence the *ldlr* knockdown exhibits increased utilization of the natural-abundance glycogen that was synthesized prior to the d-[^13^C_6_]glucose feeding).

### Glycogen quantification by combined solid-state and FTIR

**Figure 8:**
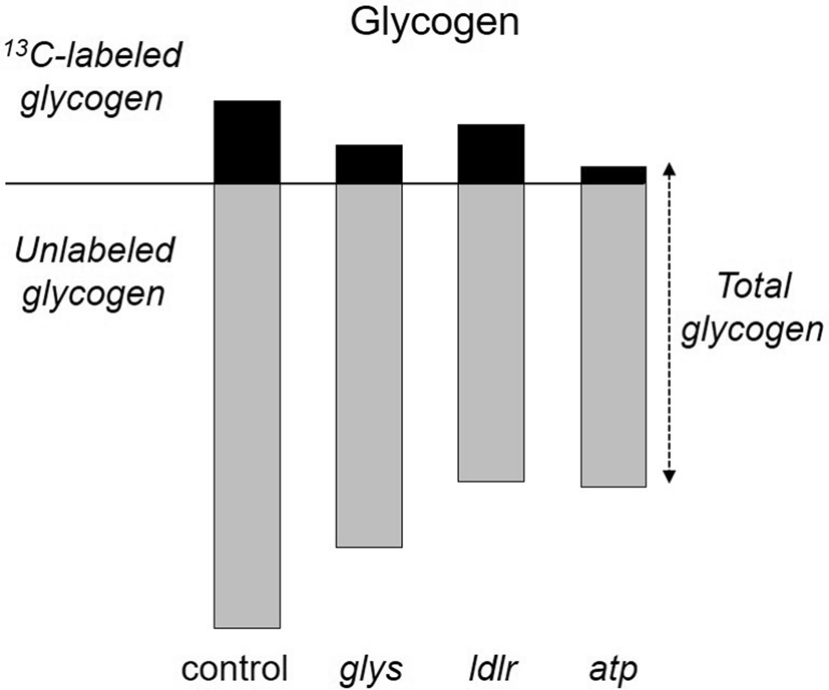
Combined NMR and FTIR quantification of glycogen. The black bars are proportional to the glycogen that was synthesized from the d-[^13^C_6_]glucose. The gray bars represent the unlabeled glycogen that was synthesized before the feeding of d-[^13^C_6_]glucose. The sum of black and gray bars represents the total glycogen accumulations in mosquitoes as measured by FTIR.

The solid-state NMR and FTIR quantifications of glycogen accumulations in dis-RNA knockdown mosquitoes are combined and shown in Fig. 8. To combine solid-state NMR and FTIR analyses, the 73-ppm peak intensity of ^13^C-natural abundance CPMAS spectrum, shown in Fig. 2b (red), was scaled using a normalization factor to equal to the 1078 cm^−1^ absorption intensity of the FTIR spectrum of dsi-control mosquitoes (Fig. 6d). The same normalization factor was used to scale the 73-ppm peak intensity of the difference spectra of ^13^C-glucose fed mosquitoes (Fig. 2b, top). In addition, a scaling factor of 1/10 was applied to the 73-ppm peak intensity of ^13^C-glucose fed mosquitoes to compensate for the ^13^C-isotope dilution (mosquitoes were fed on 10% glucose solution containing 1% ^13^C-labeled d-[^13^C_6_]glucose). The resulting intensity, shown as black bars, is directly proportional to the amount of glycogen that was synthesized from the feeding of provisioned d-[^13^C_6_]glucose in diapause-destined females. Subtracting the ^13^C-labeled glycogen (black bar) from the total glycogen (intensity of 1078 cm^−1^ absorption band) results in a gray bar that represents the unlabeled glycogen that was synthesized prior to the d-[^13^C_6_]glucose feeding.

### Lipid and glycogen biosyntheses have strong co-dependence

The dsi-RNA injection of *glys*, *atp,* and *ldlr* genes showed varying degrees of de novo glycogen biosynthesis inhibition in diapause-destined female mosquitoes. The dsi-*atp* injection which targeted the ATP synthase showed the maximum inhibition of d-[^13^C_6_]glucose utilization for glycogen biosynthesis, 86% reduction compared to the dsi-control. This was followed by the glycogen synthase knockdown^19^ that resulted in a 59% reduction in glycogen accumulation. Finally, the LDLR knockdown which would have interfered with the uptake and trafficking of LDL showed a surprising 31% reduction in glycogen accumulation (Fig. 2c, top). In addition to the inhibition of glycogen biosynthesis, solid-state NMR measurements (Figs. 2 and 3c) determined that the dsi-RNA injected mosquitoes showed the reduced 34-ppm intensity which indicated a decrease in the amount of the stored lipids. The reduced lipid accumulation was confirmed by FTIR by the reduced CH_2_ vibrational intensities at 2956 cm^−1^, 2920 cm^−1^, and 2850 cm^−1^ (Figs. 6c and 7b). This indicated that the dsi-RNA injection not only inhibited de novo lipid biosynthesis but also increased utilization of the stored triacylglyceride that was synthesized before the feeding of d-[^13^C_6_]glucose. Hence, the knockdown of Foxo downstream genes has a direct impact on the lipid homeostasis resulting in an undesirable increase in lipid expenditure in diapausing mosquitoes. This suggests that lipid and glycogen biosyntheses have strong co-dependence where the intracellular concentration of one may affect the biosynthesis of the other. One possibility for this co-dependence between lipid and glycogen biosyntheses is that LDLR acts to deliver a signaling lipid to the mosquito brain and regulate the secretion of hormones related to glucose metabolism. For example, in Drosophila, fat-containing LDLR convey information about circulating lipid composition to sensory cells in the brain to regulate insulin signaling^20^. Blocking fat-containing molecules reduced insulin signaling and subsequently modulated glucose metabolism. Although in the early diapause period, the insulin-like peptide is suppressed, its role has not been studied like other 7 or 8 insulin-like peptides and their functional role in diapausing mosquitoes^21^. It may be interesting to examine whether this factor plays a role in mosquito diapause at low temperatures.

### Concluding remarks

A study^19^ shows that suppression of the expression of the gene encoding *glys* during the early diapause period inhibited the synthesis of glycogen and lipids accumulation in mosquitoes. Moreover, suppression of the expression of this gene reduced the survival rate of diapausing females by more than 80% in 1 month. Interestingly, in the first month of the diapause program, it was found that the lipid was not used as the main energy source, but the glycogen in the fat body was used first. It is assumed that the use of lipids is determined by sensing the remaining amount of glycogen or glucose in the fat body by a mechanism that is not yet known^22^. Inhibiting the function of the *glys* gene is another evidence that supports this hypothesis, given the same consequences of inhibiting the storage of the lipid levels in this study.

ABC transporters (*atp*) are ubiquitous in all domains of life. They share the same core structure consisting of two transmembrane regions and two soluble nucleotide-binding domains. The former domains bind and translocate substrates across lipid bilayers while the later domains bind and hydrolyze ATP^23^. This protein plays an important role in the metabolism of lipids in cells by transferring of lipids to peroxisomes. Mutations in the *atp* gene also cause cytoplasmic accumulation in unbranched and saturated long fatty acids. Accumulation indicates that the mitochondria are unable to metabolize the lipids and thereby inhibiting energy production through beta-oxidation^24^. In a previous study, different activations of genes related to lipid metabolism were found in early diapause. The genes in beta-oxidation metabolism are strongly inhibited in this period, where the diapausing females need to store lipids. In contrast, after exhausting glycogen, the genes in beta-oxidation were up-regulated which include acetyl-coA synthetase, carnitine o-octanoyltransferase, acyl-coA dehydrogenase, 3-hydroxyacyl-coA dehydrogenase, and β-ketoacyl-coA thiolase. The female began to use the stored lipids, following the glycogen exhaustion, by converting the lipids into free fatty acids and then transferring them to mitochondria and peroxisomes for necessary energy metabolism^21^.

In Drosophila, the low-density lipoprotein receptor chaperone is an evolutionary conserved endoplasmic reticulum protein involved in the folding, trafficking, and quality control of LDLR proteins^6^. Since the LDLRs mediate the endocytosis of low-density lipids and the recycling of LDLRs is an essential mechanism for delivery of lipids to cells, it is speculated that RNAi suppression of the activity of the *ldlr* gene will inhibit the metabolism of the lipids in the cell. This speculation was confirmed in this study, and it was shown that suppression of the activity of the *ldlr* gene inhibits not only fat but also the metabolism of glycogen in the diapausing females (Figs. 3 and 6).

Since the diapausing mosquitoes do not feed on other nutrients during the winter, efficient consumption of the stored nutrient is important for survival during the cold winter. In addition, the stored nutrient is also used as an important resource for egg production at the end of diapause. Thus, genetic and biochemical mechanisms to control the nutrient stores and efficient use of the energy resources is important for the overwintering survival in the mosquitoes. To this end, a model has been proposed for genetic mechanisms related to efficient use and storing energy in diapause program^3,25^. The suppression of the insulin signal causes the Foxo to become active in the fat body cells and activate the downstream genes, which initiates the alternative development program (diapause) in the adult females of *Cx. pipiens*. Three of the downstream genes of the Foxo signaling are proposed in the regulation of diapause energetics and are an essential factor in synthesis, storage, and transfer of glycogens and lipids. In this study, we have shown that the genes encoding glycogen synthase (*glys*), ATP-binding cassette transporter (*atp*), and the low-density lipoprotein receptor chaperone (*ldlr*), that are the targets of Foxo transcription factor, play an important role in the energy homeosis in the mosquitoes destined for overwintering diapause.

## Materials and methods

### Insect rearing

The detailed rearing of a stock colony of *Cx. pipiens* can be found elsewhere^3^, but briefly, the *Cx. pipiens* colony was established in September 2000 from larvae collected in Columbus, OH, and additional field-collected mosquitoes were added to the laboratory colony in 2009. The colony was reared at 25 °C and 75% relative humidity under a 15-h light:9-h dark (L:D) photoperiod as previously described. When larvae reached the second instar, rearing containers were placed under one of two environmental conditions: nondiapausing females were generated by rearing at 18 °C, 75% relative humidity, and 15:9 L:D. To induce diapause, mosquitoes were reared at 18 °C, 75% relative humidity, and 9:15 L:D. To confirm diapause status, primary follicle and germarium lengths were measured, and the stage of ovarian development was determined according to the methods described by Christophers^26^.

### Synthetic dicer-substrate siRNA (dsi-RNA) injection into adult female mosquitoes

Targeting of the genes encoding glycogen synthase (*glys*, vectorbase gene i.d.: CPIJ005086), ATP-binding cassette transporter (*atp,* CPIJ012364) and low-density lipoprotein receptor chaperone (*ldlr*, CPIJ010816) was performed as described previously^3,4^. Briefly, the DsiRNAs were used in silencing experiments against these genes include dsi-glys, dsi-atp and dsi-ldlr that correspond to target sequences. The sequences of these siRNA duplexes, which were purchased from Integrated DNA Technology (IDT, Coralville, IA) and confirmed through BLAST searches to have no significant homology to *Cx. pipiens* genes other than *glys, atp* and *ldlr*, are as follows: dsi-*glys*: 5′-rArGrCrGrArCrUrCrCrArCrGrUrUrGrArArGrUrUrGrUrUrGrGrUrU-3′/5′-rCrCrArArCrArArCrUrUrCrArArCrGrUrGrGrArGrUrCrGCT-3′, dsi-*atp*: rGrArGrGrArArArUrCr CrGrArCrGrGrArCrUrGrUrArGrUrGrCrUrC-3′/5′-rGrCrArCrUrArCrArGrUrCrCrGrUrCrGrGrArUrUrUrCrCTC-3′, dsi-*ldlr*: 5′-rCrArUrArArArGrArUrGrGrCrUrCrGrArUrUrGrUrCrArUrCrGrArU-3′/5′-rCrGrArUrGrArCrArArUrCrGr ArGrCrCrArUrCrUrUrUrATG-3′. All data were confirmed following knockdown with dsi-*glys*, dsi-*atp* and dsi-*ldlr*, suggesting that none of the phenotypes reported herein were the result of off-site targeting by the dsiRNA. A scrambled negative control dsiRNA, a dsi-control duplexes lacking significant sequence homology to any genes in the *Cx. pipiens* genome, was used for control experiments, dsi-control: 5′-rGrArArGrArGrCrArCrUrGrArUrArGrArUrGrUrUrArGrCGT-3′ /5′-rArCrGrCrUrArArCrArUrCrUrArUrCrArGrUrGrCrUrCrUrUrCrCrG-3′. None of the results reported were observed in control-injected females, which were not significantly different than wild type females for any of the phenotypes assessed. Dsi-control injected and WT females in the results represent the natural abundance of the RNA transcript levels and nutrient composition.

### Dsi-RNA efficiency evaluation by qRT-PCR

Total RNA samples were extracted with TRIzol (Invitrogen) from three batches of 8–12 adult female mosquitoes on 7–10 days after dsi-RNA injection. Only adult females were kept in 12″ × 12″ × 12″ mesh cages with unrestricted access to clean water and a 10% glucose solution with or without 1% ^13^C-labeled d-[^13^C_6_]glucose. cDNA was synthesized using a High-Capacity cDNA Archive Kit (Applied Biosystems). Real-time PCR reactions were performed on a 7300 Real-Time PCR System (Applied Biosystems) using SYBR green supermix (BioRad). Reactions were run in triplicate using three independent biological replicates of each sample. Primer pairs to *glys, atp*, *ldlr,* and ribosomal protein large subunit 19 (*rpl19*, a loading control) yield single peaks in the dissociation curve as described in previous study^27,28^. mRNA expression levels of *glys*, *atp,* and *ldlr* were determined relative to *rpl19* expression by relative quantification. The following qRT-PCR primers sets were used: qRT-*glys*, CGATCCACGAGTTCCAGAAT, and GCGTCTTCTCCAGGTCAAAG; qRT-*atp*, ATCCGACGGACTGTAGTGCT, and AGGGTCAGTGCATTTTCACC; qRT-*ldlr*, AATGTTTTCCGGATTGCTTG, and CTGGATCAATGGGAGGAAGA; qRT-*rpl19*, CGCTTTGTTTGATCGTGTGT, and CCAATCCAGGAGTGCTTTTG. Statistical significance of differences in transcript levels was determined using a Student’s *t* test between the relative transcript values of dsi-*glys*, dsi-*atp*, or dsi-*ldlr* injected vs. control samples (dsi-control injected), using three biologically independent replicates for each gene (three groups of 8–12 RNAi-injected females). A *P* value of less than 0.05 was considered a significant transcript-level change.

### ^13^C-isotope labeling of ***Cx. Pipiens***

Dsi-RNA injected female *Cx. pipiens* after adult eclosion were fed for 7 days on sponges soaked with 10% glucose solution containing 1% ^13^C-labeled d-[^13^C_6_]glucose. Uniformly ^13^C-isotope labeled d-[^13^C_6_]glucose (isotopic enrichment of 99%) was purchased from Cambridge Isotope Laboratories, Inc. (Andover, MA). After the 7-days feeding, the mosquitoes were frozen at − 80 °C then lyophilized for 3 days. Lyophilized intact mosquitoes were weighed and packed into a 3.2-mm zirconia rotor for solid-state NMR analysis^12,19^.

### Solid-state NMR

Solid-state ^13^C cross-polarization magic-angle spinning (CPMAS) NMR^29^ of diapause-destined female *Cx. pipiens* injected with dsi-RNA and then fed for 7 days with 10% glucose solution containing 1% ^13^C-labeled d-[^13^C_6_]glucose was collected on 11.75-T (proton radio frequency of 500 MHz) Bruker Avance NEO with a double resonance HX probe. ^13^C-CPMAS NMR was performed as described earlier^12,19^. Briefly, lyophilized mosquitoes were contained in a 3.2-mm outer diameter zirconia rotor with Kel-F endcap spinning at 10 kHz. Proton-carbon matched cross-polarization ramp was at 50 kHz with 2-ms contact time. The proton dipolar decoupling was achieved by applying continuous-wave spinal64^30^ on the ^1^H channel during acquisition. The π pulse length was 2.5 µs for ^1^H and the recycle delay was 5 s. The line broadening for the spectrum was 50 Hz. The spectra were normalized to equal 175-ppm intensity of peptidyl-carbonyl carbons in proteins.

### FTIR

FTIR spectra were obtained using Thermo Scientific Nicolet iS50 Spectrometer with iS50 ATR module containing a diamond crystal (Madison, WI, USA), equipped with deuterated triglycine sulfate detector working at room temperature. Spectra acquisition was performed in the 500–4000 cm^−1^ range with 4 cm^−1^ resolution, after 100 scans, using the N-B Strong as apodization and 2-levels zero fillings of the interferogram giving data spacing of 0.482 cm^−1^; these were converted into absorbance using Thermo Scientific OMNIC software version 9.2 (https://www.thermofisher.com/order/catalog/product/INQSOF018#/INQSOF018). Powdered lyophilized mosquito samples, following the solid-state NMR measurements, were removed from the zirconia rotor, and then measured directly without any further preparation. Background measurement of air was taken and automatically subtracted from the sample measurements. Spectra were acquired from five randomly selected locations across the sample to minimize sampling bias. Between measurements, the ATR crystal was carefully cleaned using ethanol (Sigma-Aldrich, analytical standard) and dried with light-duty tissue wipers. Using the same method of analysis, FTIR spectra of canola oil, glycogen from bovine liver (Sigma-Aldrich), bovine serum albumin (BSA) (lyophilized powder, Sigma-Aldrich), deoxyribonucleic acid sodium salt from salmon testes (Sigma-Aldrich), and chitin from shrimp (Sigma-Aldrich) were obtained. These were used as standards for the identification of absorption peaks for lipids, glycogen, protein, DNA, and chitin, respectively.

## Abbreviations

ATR-FTIR: Attenuated total reflection Fourier-transform infrared spectroscopy
CPMAS: Cross-polarization magic-angle spinning
Glc: Glucose
dsi-RNA: Synthetic dicer-substrate siRNA
Foxo: Forkhead of transcription factors
GlcNAc: *N*-Acetylglucosamine
NMR: Nuclear magnetic resonance
TAG: Triacylglyceride
